# Test and re-test reliability of optimal stimulation targets and parameters for personalized neuromodulation

**DOI:** 10.3389/fnins.2023.1153786

**Published:** 2023-05-12

**Authors:** Feng Fang, Jared Cammon, Rihui Li, Yingchun Zhang

**Affiliations:** ^1^Department of Biomedical Engineering, University of Houston, Houston, TX, United States; ^2^Department of Psychiatry and Behavioral Sciences, Center for Interdisciplinary Brain Sciences Research, Stanford University, Stanford, CA, United States

**Keywords:** optimal neuromodulation, test–retest reliability, brain controllability, optimal control analysis, DTI, resting fMRI

## Abstract

Protocols have been proposed to optimize neuromodulation targets and parameters to increase treatment efficacies for different neuropsychiatric diseases. However, no study has investigated the temporal effects of optimal neuromodulation targets and parameters simultaneously via exploring the test–retest reliability of the optimal neuromodulation protocols. In this study, we employed a publicly available structural and resting-state functional magnetic resonance imaging (fMRI) dataset to investigate the temporal effects of the optimal neuromodulation targets and parameters inferred from our customized neuromodulation protocol and examine the test–retest reliability over scanning time. 57 healthy young subjects were included in this study. Each subject underwent a repeated structural and resting state fMRI scan in two visits with an interval of 6 weeks between two scanning visits. Brain controllability analysis was performed to determine the optimal neuromodulation targets and optimal control analysis was further applied to calculate the optimal neuromodulation parameters for specific brain states transition. Intra-class correlation (ICC) measure was utilized to examine the test–retest reliability. Our results demonstrated that the optimal neuromodulation targets and parameters had excellent test–retest reliability (both ICCs > 0.80). The test–retest reliability of model fitting accuracies between the actual final state and the simulated final state also showed a good test–retest reliability (ICC > 0.65). Our results indicated the validity of our customized neuromodulation protocol to reliably identify the optimal neuromodulation targets and parameters between visits, which may be reliably extended to optimize the neuromodulation protocols to efficiently treat different neuropsychiatric disorders.

## 1. Introduction

Neuromodulation is effective in treating different neuropsychiatric disorders such as Parkinson’s disease (PD) and Depression (Horn and Fox, 2020). For example, repetitive transcranial magnetic stimulation (rTMS) has shown effectiveness as a non-invasive neuromodulation tool to treat depression, which has been approved by the FDA (Perera et al., 2016). Nevertheless, current neuromodulation outcomes are highly variable due to patient heterogeneity. Meanwhile, the utilization of empirical stimulation targets and parameters for treating different individuals further reduces the possibility to accommodate the heterogeneity. Thus, previous studies have proposed optimized and personalized neuromodulation protocols to consider patient heterogeneity (Cole et al., 2021; Modak and Fitzgerald, 2021; Klooster et al., 2022). For example, prior study utilized the resting-state functional magnetic resonance imaging (fMRI) signal to locate the optimal stimulation target inside the dorsolateral prefrontal cortex (DLPFC) and change the stimulation intensities according to the depth of the stimulation location to ensure delivering of 90% resting motor threshold (RMT) for personalized neuromodulation treatment (Cole et al., 2021). However, even though the stimulation target is optimized, the target is still constrained within the DLPFC, and the stimulation parameters like frequency, duration, and amplitude, were not optimized for specific brain states transition. To maximize the neuromodulation efficacy for treating various diseases, it is important to personalize the neuromodulation protocols by simultaneously optimizing the stimulation targets and parameters across the whole brain for specific brain states transition. In addition, it is also important to ensure the stability of the optimal neuromodulation protocols for each individual subject across visits to reduce the time needed for treatment, reduce and costs, and properly interpret the results (Jannati et al., 2019).

Recently, network control theory has been proposed to optimize the neuromodulation targets in human brain (Gu et al., 2015). Brain network controllability has been developed to characterize the brain’s dynamic properties, assuming the brain system is deliberately shifted or guided along a particular trajectory to support specific goals (Gu et al., 2015; Fang et al., 2021, 2022a,b). As such, it provides a potentially powerful tool for modeling the neuromodulation process in human brain (Tang and Bassett, 2018). Previous studies have illustrated that the brain controllability measurements can predict the effects of external stimulation on the neural activations, where the brain dynamics are identified by the structural brain connectivity network via the diffusion tensor imaging (DTI) (Bassett and Sporns, 2017; Medaglia et al., 2018; Tang and Bassett, 2018; Fang et al., 2021). For example, previous study has shown that the stimulation of high controllability brain region will easily move the brain network system to nearby brain states with little control energy (Muldoon et al., 2016). Meanwhile, another study utilizes controllability measures to assess the TMS effects on language performance and illustrates that brain network controllability in the inferior frontal gyrus (IFG) is related to controlled language variability and susceptibility to TMS (Medaglia et al., 2018). Together, these results indicate that brain controllability measurements are potential biomarkers for determining optimal stimulation targets for optimal neuromodulation in clinical practice.

To optimize the neuromodulation parameters, a potential framework for such optimization is optimal control analysis. Optimal control analysis deals with the calculation of optimal parameters that can steer the brain network dynamic to a specific state (Ross, 2015). The purpose of optimal control analysis is to minimize the difference between the initial state and the expected target state, and the control energy necessary to steer the brain between various states through a period of time (Stiso et al., 2019). Previous study has demonstrated that the optimal control analysis could predict the effect of direct electrical stimulation in improving the memory encoding (Stiso et al., 2019). In addition, a recent study has reported that the optimal control analysis can infer the optimal control energy needed for seizure control in patients with epilepsy (Scheid et al., 2021). The results indicated that the control energy to steer the brain from an ictal brain state to a seizure-free state is smallest during seizure onset.

Although optimal neuromodulation protocols have been proposed by incorporating the network control theory and optimal control analysis, whether the optimized stimulation targets and parameters exhibit good test–retest reliability is still unclear. Test–retest reliability measure is important for the inference of convincing conclusions and serve as potential clinical biomarkers (Elliott et al., 2020). Currently, as the employment of plasticity-induced neuromodulation protocols becomes more popular, increasing attention has been given to the test–retest reliability studies using structural and functional MRI data to design optimal neuromodulation protocols (Fox et al., 2013; Rosenstock et al., 2017; Ning et al., 2019). Test–retest reliability of the optimal neuromodulation protocols may be potentially affected by several factors such as the stimulation targets and the stimulation parameters between visits. However, no systematic study has been performed to investigate the test–retest reliability of neuromodulation protocols by simultaneously considering the test–retest reliability of the optimal stimulation targets and parameters based on brain controllability and optimal control analysis.

In this study, we analyzed a recently public structural and functional MRI dataset, which included 57 healthy young subjects who was each scanned twice around 6 weeks apart. We first located the optimal stimulation targets for each subject based on the brain controllability analysis using DTI data scanned from the two visits, respectively, and assessed the test–retest reliability of the optimal stimulation targets. Then, we calculated the optimal stimulation parameters to steer the resting brain state from visit 1 to visit 2 based on optimal control analysis and evaluated the test–retest reliability of the optimal stimulation parameters and the model fitting accuracies between the actual final state and the simulated target state. This work offers a systematic support for assessing the test–retest reliability of optimal neuromodulation protocols by considering the reliability of both the stimulation targets and parameters, providing reliability for future clinical applications.

## 2. Materials and methods

### 2.1. Participants

Repeatedly measured DTI and resting-state fMRI (rs-fMRI) data were obtained from the Connectivity-based Brain Imaging Research Database (C-BIRD) at Beijing Normal University (Lin et al., 2015). Fifty-seven healthy young adults (M/F: 30/27, age 23.05 ± 2.29 years) underwent repeated MRI scans in two visits with an interval of around 6 weeks (40.94 ± 4.51 days) between two visits. Seven subjects were excluded due to the loss of rs-fMRI data in the open-source dataset. Written informed consent was given by each participant, and the study was approved by the Institutional Review Board of the State Key Laboratory of Cognitive Neuroscience and Learning at Beijing Normal University. All participants were right-handed, native Chinese speakers and had no history of neurological or psychiatric disorders.

### 2.2. Data acquisition

The instruction before and after MRI scans is shown in Table 1. All MRI data were obtained using a SIEMENS Trio Tim 3.0 T scanner (Siemens Healthcare, Erlangen Germany) with a 12-channel phased-array head coil in the Imaging Center for Brain Research, Beijing Normal University (Lin et al., 2015). Structural MRI data were acquired using a T1-weighted, sagittal 3D magnetization prepared rapid gradient echo (MP-RAGE) sequence. Diffusion weighted imaging data were acquired using a single-shot twice-refocused spin-echo diffusion echo-planar imaging (EPI) sequence with implementation of the parallel imaging scheme named GeneRalized Autocalibrating Partially Parallel Acquisitions. The rs-fMRI data were obtained using a T2*-weighted echo-planar imaging (EPI) sequence. The detailed acquisition parameters can refer to (Lin et al., 2015).

**Table 1:** Instructions before and after MRI scan.

(a)	Do not undertake strenuous exercise on the day before the fMRI scan
(b)	Do not consume hard drinks on the day before the MRI scan
(c)	Do not consume stimulating drinks for 6 h before the MRI scan
(d)	Have a good rest on the day before the MRI scan to ensure good conditions for scanning
(e)	Lie still to rest and relax, keep motionless as possible during MRI scan
(f)	Keep eyes closed but do not fall asleep (for resting-state fMRI scan)
(g)	Ask the subjects whether they kept their eyes closed or fall asleep during the scan

### 2.3. Data preprocessing

Preprocessing of DTI data was performed using DSI Studio toolbox.^1^ Image distortion and motion artifact induced by the eddy currents were corrected to reduce the noisy components. Q-space diffeomorphic reconstruction (QSDR) method was then utilized to reconstruct the DTI data (Yeh and Tseng, 2011). The diffusion-weighted images in native space were first reconstructed and the quantitative anisotropy (QA) in each voxel was calculated. The computed QA values were then utilized to warp the brain to a template QA volume in MNI space using the SPM non-linear registration method (Tzourio-Mazoyer et al., 2002), where the spin density functions were reconstructed. Fiber tracking was then conducted with the following parameters: angular cutoff = 90, step size = 1.0 mm, minimum length = 10 mm, spin density function smoothing = 0.0, maximum length = 800 mm. A modified FACT algorithm was then utilized to perform deterministic fiber tracking until 100,000 streamlines were reconstructed for each individual subject.

Preprocessing of resting-state fMRI data was perform using the SPM12-based DPABI toolbox (Yan et al., 2016). First 10 time points were removed to keep magnetization equilibrium. The preprocessing steps included spatial normalization, slice-time correction, spatial smoothing, head motion correction, and linear trend removal. A temporal bandpass filter from 0.01 to 0.08 T was then used to filter the low-frequency drift, cardiac noise, and physiological respiratory. Parcellation of the brain was performed using the Desikan-Killiany-Tourville (DKT) atlas, resulting in 62 regions of interest (ROIs) (Klein and Tourville, 2012).

### 2.4. Brain controllability analysis

Controllability represents the ability of a specific brain region in steering the state of the dynamic network system into different brain states (Gu et al., 2015). One of the critical steps to employ the brain controllability analysis is to define a structural connectivity network and the network dynamic of the brain (Gu et al., 2015). In our calculation, the structural brain network was estimated by the streamlines connecting each two-brain region constructed by the DTI data. A simplified, noise-free, and linear time-invariant network dynamic model was then be built as follows:


(1) x(t+1)=Ax(t)+Bu(t)

where x with dimension N × 1 (N is the number of brain regions) describes the brain state of different brain regions over time, and A with dimensions N × N is the weighted structural connectivity matrix as described above. The matrix B with dimension N × m represents the input matrix and the matrix u with dimension *m* × 1 represents the external stimulation. The *m* denotes the number of targeted nodes.

#### 2.4.1. Average controllability

Average controllability represents the ability of a specific brain region in steering the network dynamic system into many easy-to-reach states with minimal input energy (Gu et al., 2015). It was calculated as the $H_{2}$-norm of the network system. Mathematically, it was defined as:


(2) $$a_{c}=\mathrm{Trace}\left(\overset{\infty }{\underset{\tau =0}{\sum }}A^{\tau }B_{k}B_{k}^{T}A^{\tau }\right)$$

Cognitively, the high average controllability brain areas are critical in moving the brain to different cognitive states that require little cognitive effort.

#### 2.4.2. Modal controllability

Modal controllability represents the ability of a specific brain area in moving the brain network system into many difficult-to-reach states (Gu et al., 2015). Mathematically, it was defined as:


(3) $$\phi_{i}=\overset{N}{\underset{j=1}{\sum }}\left(1-\lambda_{j}^{2}\left(A\right)\right)v_{ij}^{2}$$

$v_{ij}$ is the element of the eigenvectors matrix of *A* and $\lambda_{j}$ is the *j*th eigenvalue. Cognitively, high modal controllability brain areas are critical in moving the brain into various cognitive states that require a lot of cognitive effort.

### 2.5. Personalization of the neuromodulation parameters via optimal control analysis

Details of parameters estimation of optimal control process can be found in (Stiso et al., 2019). Briefly, the optimization problem was first defined as:


(4) $$\min_{u}\overset{T}{\underset{0}{\int }}{\left(x_{T}-x\left(t\right)\right)}^{\prime }S\left(x_{T}-x\left(t\right)\right)+\rho u_{K}{\left(t\right)}^{\prime }u_{K}dt$$

$$s.t.\dot{x}=Ax\left(t\right)+Bu\left(t\right),x\left(0\right)=x_{0},\mathrm{and}\;x\left(T\right)=x_{T}$$

where $x_{T}$ represents the target state, *T* represents the control horizon, and ρ is to weight the input constraint. *S* represents the group of nodes to constrain.

Brain area with the maximum controllability was first selected as the optimal stimulation target, and then, optimal control analysis was applied to the stimulation target to steer the resting brain state at visit 1 to the resting brain state at visit 3 via performing the open-loop control. The maximum modal correlation between the actual final state (resting state at visit 2) and the simulated final state was then calculated to quantify the stimulation effects of the targeting strategies. The model fitting accuracy was computed by selecting the control horizon ***T*** from 0 to 0.75, with an increased step of 0.05 to the ***T*** (so ***T*** = 0.05, 0.10, 0.15, 0.20, …), and calculating the maximum model correlation within this period. The total energy necessary to steer the brain into different cognitive states can be described as:


(5) $$E_{k,x_{0}x_{T}}=\overset{T}{\underset{0}{\int }}{\left\|B_{k}u_{x_{0}x_{T}}\right\|}_{2}^{2}dt.$$

### 2.6. Reliability analysis of brain controllability and optimal parameters

Intra-class correlation (ICC) was employed to quantify the test–retest reliability of the brain controllability measurements and optimal neuromodulation parameters between visit 1 and visit 2 (Koo and Li, 2016). The ICC value was calculated based on the equation:


(6) $$\mathrm{ICC}=\frac{\mathrm{BMS}-\mathrm{WMS}}{\mathrm{BMS}+\left(m-1\right)\mathrm{WMS}}$$

where BMS(WMS) represents the between-subject (within-subject) mean square and m represents the number of repeated measurements (here, *m* = 2). We calculated the ICC values of the brain controllability measurements and the optimal neuromodulation parameters of each subject. Then, the reliability was assessed with the classifying criteria of ICC values: less than 0.4 indicated low reliability; 0.40 to 0.60 indicated fair reliability, 0.60 to 0.75 indicated good reliability, and 0.75 to 1.00 indicated excellent reliability (Koo and Li, 2016).

### 2.7. Statistical analysis

**Figure 1:**
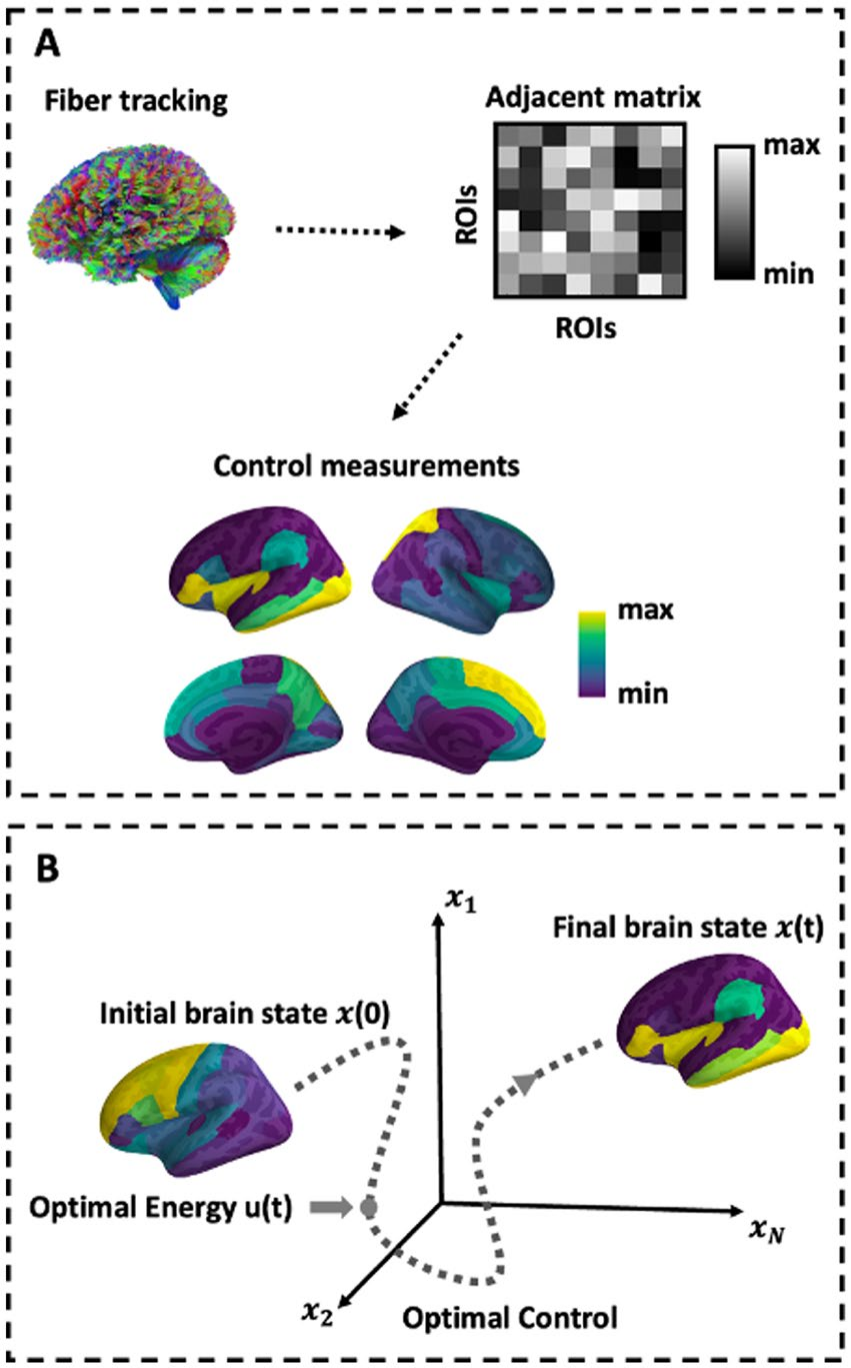
Schematic of methods. **(A)** Depiction of network construction and the control sets representation. **(B)** Schematic of the optimal control paradigm. In the optimal control design, the initial brain state *x*(0) has some position in space that evolves over time toward a predefined target state.

The average and modal controllability distribution was statistically compared, respectively, between visit 1 and visit 2 using paired *t*-test (Kim, 2015). Linear regression analysis was performed to identify the relationship of global average and modal controllability, respectively, between visit 1 and visit 2 (Draper and Smith, 1998). Similarly, the same linear regression analysis was applied to find the relationship of regional average and modal controllability, respectively, between visit 1 and visit 2. The ICC was employed to verify the test–retest reliability of the brain controllability and the optimal neuromodulation parameters between visit 1 and visit 2. The ICC was also calculated to evaluate the test–retest reliability of the brain controllability in default mode network (DMN), frontoparietal network (FPN), and cognitive control network (CCN), respectively. Paired *t*-test was performed to compare the optimal neuromodulation parameters and the model fitting accuracies between visit 1 and visit 2. Linear regression analysis was also performed to identify the relationship between the simulated target state and actual target state. Outliers, defined by mean plus/minus three standard deviations, were excluded (Howell et al., 1998). All *p* values were corrected by Bonferroni correction (Armstrong, 2014) (see Figure 1).

## 3. Results

### 3.1. Brain controllability distribution maps across visits

**Figure 2:**
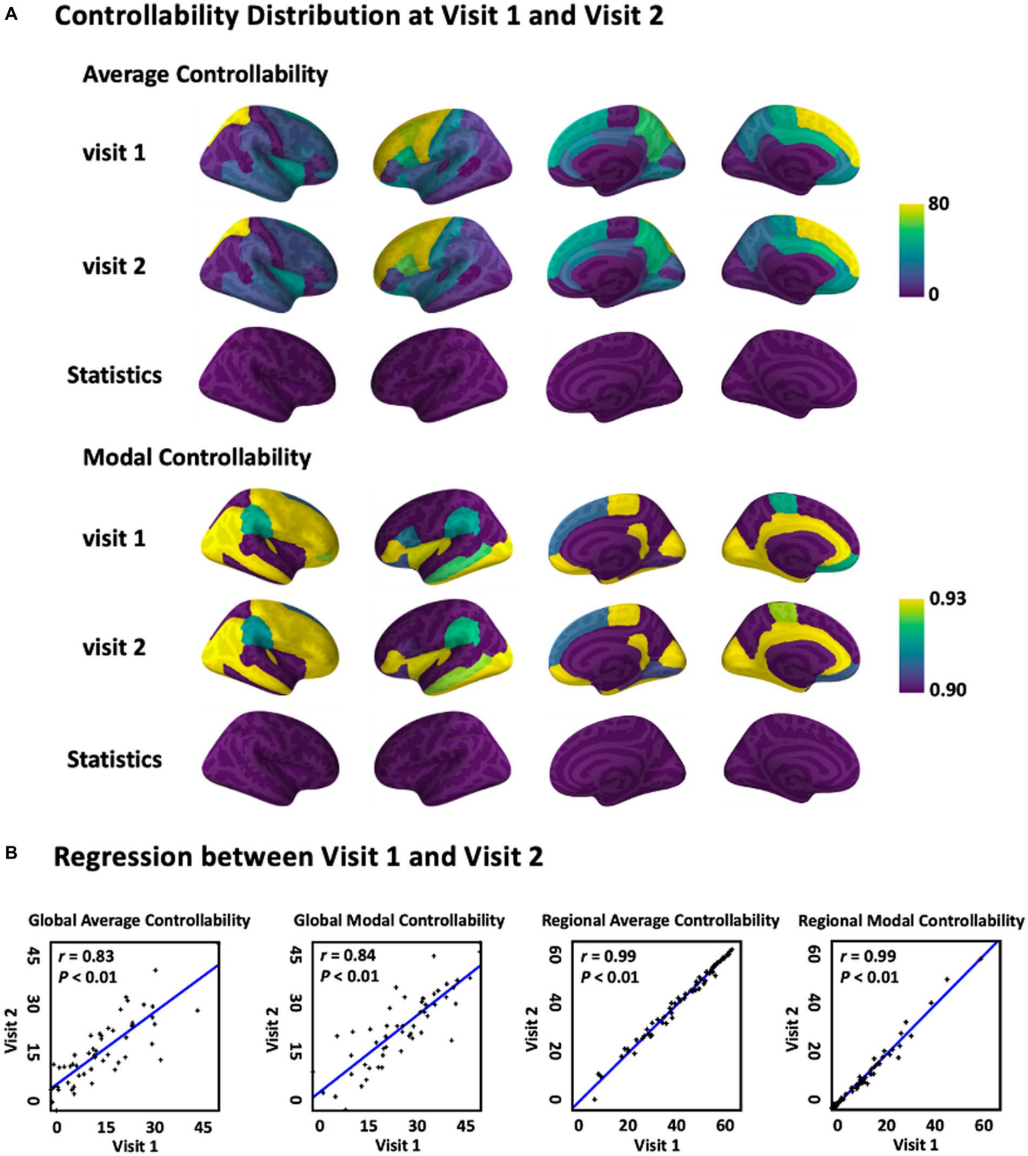
Average and modal controllability between Visit 1 and Visit 2. **(A)** Average and modal controllability distribution(averaged across subjects) and statistical comparison between Visit 1 and Visit 2. **(B)** Regression of global and regional average and modal controllability between Visit 1 and Visit 2. For global controllability, the *x* and *y* axes representeach single subject, while for regional controllability, the *x* and *y* axes represent each single brain area.

The average and modal controllability distribution over the brain averaged across subjects were first documented and statistically compared, respectively, between visit 1 and visit 2 (Figure 2A). The results indicated that there was no significant difference of both average and modal controllability measurements, respectively, derived from visit 1 and visit 2 (*p* > 0.05), indicating high stability of the controllability measurements calculated from the DTI data. Furthermore, the relationship of the controllability measurements between visit 1 and visit 2 were explored using linear regression analysis (Figure 2B). The results showed that the global average controllability and the global modal controllability at visit 1 were significantly correlated with the respective measurements at visit 2 (*r* = 0.83, *p* < 0.01), indicting the stability of the brain controllability measurements in a global scale. In addition, the results also showed that the regional average and modal controllability, respectively, were significant correlated between visit 1 and visit 2 (*r* = 0.99, *p* < 0.01). Together, these results indicated that the brain controllability measurements were stable across recording sessions.

### 3.2. Reliability of brain controllability across visits

**Figure 3:**
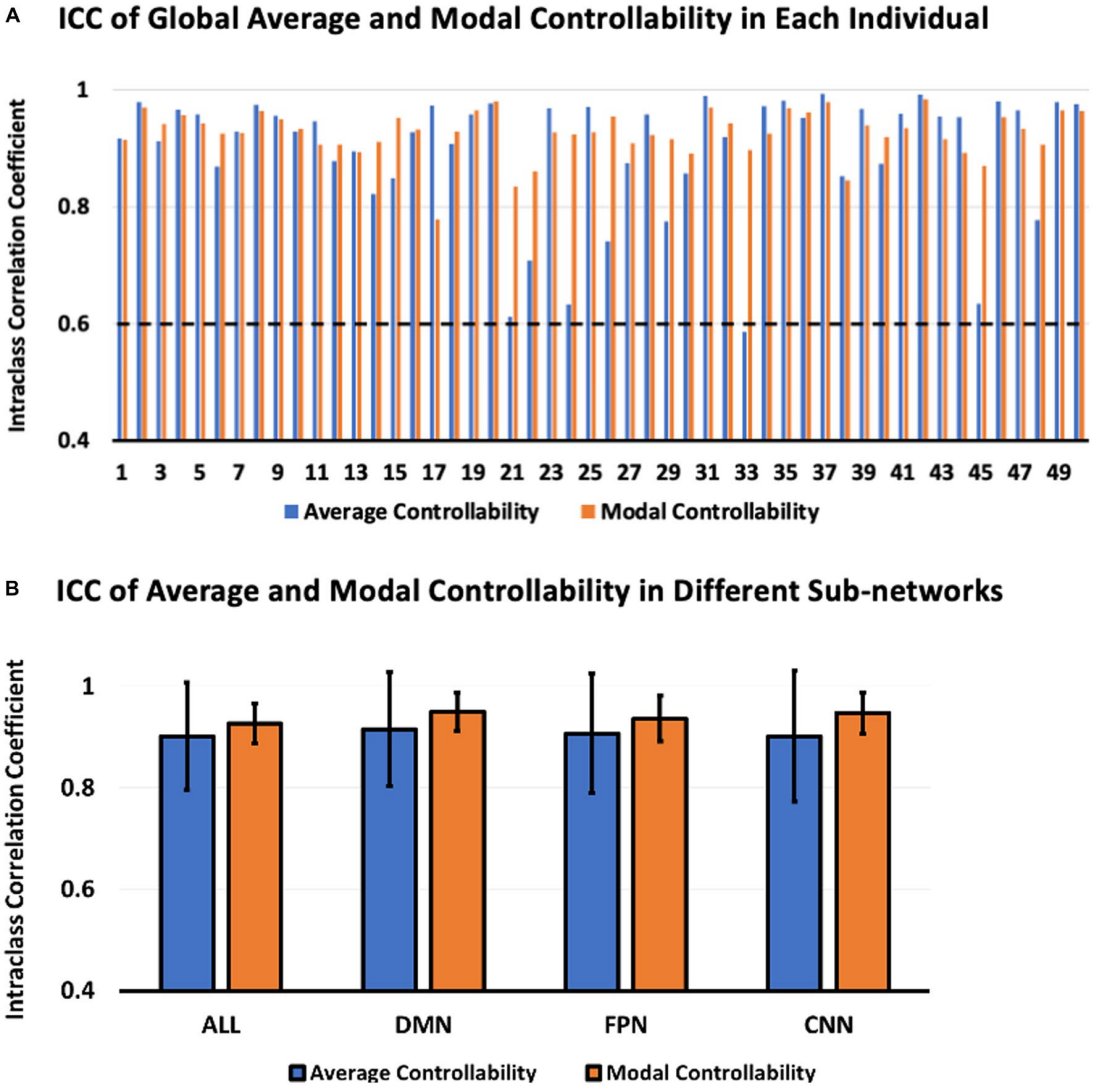
ICC of average and modal controllability between Visit 1 and Visit 2. **(A)** ICC of global average and modal controllability in each individual. **(B)** ICC of regional average and modal controllability in different brain sub-networks.

The ICC of global average and modal controllability of different individuals were shown in Figure 3A. According to the ICC classification criteria, the ICC values larger than 0.75 indicate excellent reliability. From the results shown in Figure 3A, we can see that the global average and modal controllability of most subjects were higher than 0.8, indicating the excellent test–retest reliability of the global average and modal controllability measures. The results shown in Figure 3B demonstrated the ICC of average and modal controllability measurements within three human brain psychological sub-networks, including DMN, FPN, and CNN. The results indicated that the test–retest reliability of controllability measurements in these critical sub-networks were also excellent. Together, these results indicated that the test–retest reliability of global and regional controllability measurements were excellent in various individual subjects.

### 3.3. Reliability of optimal neuromodulation parameters

**Figure 4:**
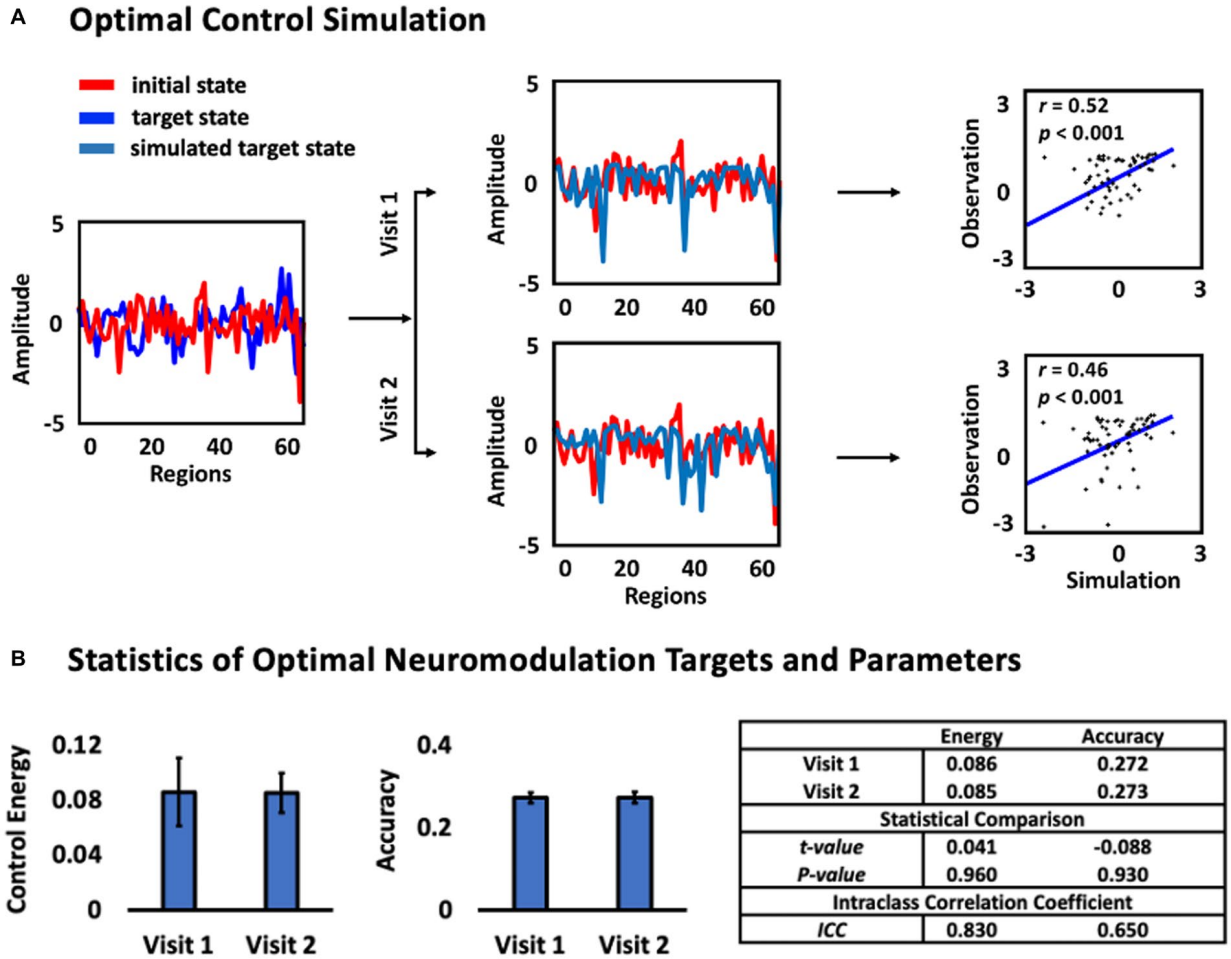
Optimal control analysis. **(A)** Optimal control simulation. The first column represents the initial state (blue line) and target state (red line). The second column represents the fitting between the simulated final state and (dark blue line) the actual final state (red line) based on two different structural brain networks, respectively. The third column represents the regression results between the simulated target state (Simulation) and the actual target state (Observation). **(B)** Statistics of the optimal neuromodulation targets and parameters between Visit 1 and Visit 2.

The results in Figure 4A shows an example of the optimal control simulation based on the structural brain networks constructed from the DTI data recorded from visit 1 and visit 2, respectively. The first column shows the fitting curve between the resting brain state at visit 1 (initial state, blue color) and the resting brain state at visit 2 (actual final state, red color). The resting brain state was represented by the *z*-scored mean of rs-fMRI signal amplitudes over times at each region of interest (ROI). The second column shows the fitting curve between the actual final state (red line) and the simulated final state (dark blue line) after exerting the optimal neuromodulation parameters to the optimal neuromodulation targets based on the structural brain network constructed from visit 1 and visit 2, respectively. The third column shows the regression results to find the relationship between the Observation (actual final state) and the Simulation (simulated final state). Results showed that the Observation and the Simulation curves were fitted well, and all significantly correlated no matter which structural brain network based on (based on the structural brain networks at visit 1: *r* = 0.52, *p* < 0.001, visit 2: *r* = 0.46, *p* < 0.001). Meanwhile, the ICC of the optimal neuromodulation parameters and accuracies were computed (Figure 4B). The results showed that there was no significant difference of the optimal neuromodulation parameter, including optimal neuromodulation parameters (*p* = 0.96), which was the optimal control energy calculated, and accuracy (*p* = 0.93) between visit 1 and visit 2. The ICC of the optimal neuromodulation parameters was 0.83 and the accuracy was 0.65, indicating excellent and good reliability according to the classification criteria of ICC.

## 4. Discussion

Test–retest reliability of neuromodulation measurements influence their utility as potential neurophysiologic biomarkers for therapeutic intervention. As the use of plasticity-induced neuromodulation protocols become more common, it is necessary to investigate the reliability of the optimal stimulation targets and parameters derived from the brain network system. Therefore, this study proposed a comprehensive investigation of our customized optimal neuromodulation protocols by assessing the test–retest reliability of the optimal stimulation targets and the parameters derived from the brain controllability analysis and optimal control analysis, respectively. Our results suggested that the global controllability and regional controllability of DMN, FPN, and CNN exhibited excellent reliability with all ICC values larger than 0.80. Meanwhile, our results also demonstrated that the optimal neuromodulation parameters also showed excellent test–retest reliability with ICC value larger than 0.83. Finally, our results showed that the model fitting accuracies between the actual final state and the simulated final state displayed good test–retest reliability with ICC value larger than 0.65. Together, these results indicated the reliability of our proposed neuromodulation protocol to be employed in future clinical practice.

Brain controllability analysis on structural brain network predict the capability of the brain to alter large-scale neural circuit, assuming the structural brain networks can modulate the transition between brain states (Gu et al., 2015). Specifically, the average and modal controllability diagnostics are the measurements to assess the ability of the brain to enter various cognitive states. Average controllability is to quantify the nodes’ capability to move the brain network system into many easily reachable state, while the modal controllability is to evaluate the nodes’ ability to steer the brain network system into different difficult-to-reach cognitive states. If the control energy can represent the cognitive efforts and the brain states can represent the cognitive functions, then the easily reachable states refers to the brain states that require little cognitive effort to reach, while the difficult-to-reach states represent the brain state that needs lots of cognitive effort to enter (Gu et al., 2015).

As reported by a previous study, the stimulation of brain regions with high average controllability contributes to easily steering the brain network system into nearby brain network states with little control energy (Muldoon et al., 2016). In addition, previous study has also employed the brain controllability measurements to identify the relationship between the regional controllability and the controlled language variability and susceptibility to TMS and found that a statistic that quantified the IFG’s theoretically predicted control of difficult-to-reach states explained vulnerability to TMS in the closed-ended response task (Medaglia et al., 2018). These studies, together, indicate that the brain controllability measurements are useful to design the neuromodulation protocols by defining the optimal stimulation targets for modulating various neurophysiological behaviors. In our study, we went even further to investigate the test–retest reliability of the brain controllability measurements to reliably select the optimal stimulation targets for later optimal control analysis. Our results showed that the test–retest reliability of global average and modal controllability, and the regional average and modal controllability, especially the brain areas located in DMN, FPN, and CNN, were excellent with all the ICC values larger than 0.8. DMN, FPN, and CCN are three distinct brain networks that play important roles in controlling brain function. Specifically, DMN plays a crucial role in regulating and controlling brain activity during rest and introspection. It is thought to be involved in self-referential thinking, episodic memory, and social cognition. The DMN also plays a role in regulating the level of arousal and attention, as it is active during states of mind-wandering and relaxation. Differently, FPN plays a key role in controlling attentional processes. It is responsible for maintaining and manipulating information in working memory and for directing attention to relevant information. The FPN is also involved in decision-making, planning, and problem-solving. Besides, CCN is involved in the control and regulation of cognitive and emotional processes. It plays a role in attentional control, response inhibition, and conflict resolution. The CCN also helps regulate the emotional responses to stimuli and to modulate emotional processing. These three networks work together to regulate and control brain function, supporting a wide range of cognitive and perceptual processes. Dysfunction in any of these networks has been linked to a range of neuropsychiatric disorders, highlighting their significance in brain function and disease. Hence, it is of utmost importance to study the test–retest reliability of DMN, FPN, and CCN networks in healthy individuals, which will aid in future exploration of neural control pattern changes in patients with different neuropsychiatric conditions. These results provide confidence for future clinical application to employ the brain controllability measurements for reliably determining the optimal stimulation targets.

While brain neuromodulation has great therapeutic potential, its optimization remain challenging, in part due to a lack of understanding of how stimulation affects different brain states of both neighboring and distant brain areas (Stiso et al., 2019). Recently, optimal control analysis has already been applied to optimize the neuromodulation parameters for each individual patient to steer the brain states from an initial state to an expected targeted state over a period of time (Stiso et al., 2019). Previous studies have quantified the effectiveness of the optimal control parameters inferred by the optimal control analysis to predict the influence of direct electrical stimulation to improve the performance of memory encoding. Besides, it has been employed to compute the optimal control energy necessary for seizure control in patients with epilepsy (Scheid et al., 2021). Again, in our study, we went even further to assess the test–retest reliability of the optimal stimulation parameters calculated by the optimal control analysis. Our results showed that the optimal stimulation parameters also achieved excellent test–retest reliability with the ICC value larger than 0.8, indicating that we can employ the optimal control analysis to reliably compute the optimal neuromodulation parameters based on the structural brain networks constructed from different visits. Besides, we also calculated the model fitting accuracies between the simulated final state and actual final state and demonstrated that the model fitting accuracies could achieve a good reliability with the ICC value larger than 0.65. Together, these results indicated the feasibility and the excellent reliability of our optimal neuromodulation protocol across visits.

In this study, the term “stimulation parameter” refers to the control energy, which can be further decomposed into different parameters like stimulation intensity and frequency for external stimulation devices such as rTMS and tACS used in clinical practice. Therefore, in clinical applications, we can initially determine the stimulation target based on brain controllability analysis and fix the stimulation frequency, such as 10 Hz for depression treatment using rTMS. Next, we can optimize the time-varying stimulation intensity using optimal control analysis for neuromodulation in patients. Recent advancements in neuromodulation devices, such as the Soterix Medical M×N 33/65 High Definition-transcranial Electrical Stimulator,^2^ enable automatic generation of stimulation waveform via any stimulation electrode combination. Hence, we believe that our proposed brain controllability and optimal control analysis, coupled with auto-generation of waveform technology, will improve the efficacy and accuracy of neuromodulation while reducing its complexity in clinical applications.

To the best of our knowledge, this study represents the first effort to assess the test–retest reliability of the neuromodulation protocol by simultaneously considering the test–retest reliability of the optimal stimulation targets and the optimal stimulation parameters inferred from the brain controllability analysis and optimal control analysis, respectively. There are certain limitations existed in this study. First, our proposed brain controllability analysis and optimal control analysis rely on particular assumptions and constraints such as the linearity and stationarity of the network. Failing to consider non-linear and non-stationary effects may lead to inaccurate predictions and may obscure important features of the network. These limitations need further improvement and validation in future research to enhance the accuracy of identifying optimal stimulation targets and parameters. In addition, a more fine-grained parcellation atlas should be utilized in future studies to reflect both functional and structural features of the brain (e.g., the Glasser, Schaefer, or Brainnetome atlas) (Schaefer et al., 2018), which will be helpful to improve the accuracy and specificity of brain mapping, enhance the producibility of findings across different studies, and aid the development of personalized medicine. Secondly, this study only includes healthy young participants, limiting the generalization of the results. Future research will include a broader range of participant groups with patients of different age, genders, and disease phenotypes, to better understand the application of our proposed personalized neuromodulation strategy in treating various neuropsychiatric disorders. Additionally, in this study, we described the brain state using the *z*-scored mean of the amplitude of resting state fMRI signals, however, it can also be described by other representations such as the power, entropy, and graph measures (Whitten et al., 2011; Li et al., 2019; Fang et al., 2020; Kang et al., 2021). Besides, the effectiveness of neuromodulation interventions can be influenced by various factors such as individual differences, region-specificity, stimulation parameters, and stimulation patterns. To comprehensively evaluate the intervention effects, future research will consider these factors since they may significantly impact the reliability and effectiveness of neuromodulation interventions. Finally, the initial state and target state defined in this study were both resting brain states described by the resting state fMRI signals. This may not be able to fully reveal the advantage of our neuromodulation protocol in steering any two brain states transition. However, this will be improved in future studies when we record both resting and task-induced fMRI signals such as working memory, motor performance, and other cognitively demanding tasks from the same subjects. The proposed personalized neuromodulation approach will be validated by other neuroimaging modalities such as functional near-infrared spectroscopy (fNIRS) and electroencephalogram (EEG) to design more portable and costless neuromodulation protocols (Chung et al., 2015; Li et al., 2022a,b). In this study, we proposed a personalized neuromodulation protocol, which can be easily implemented into different brain stimulation devices like deep brain stimulation and direct electrical stimulation to treat various diseases such as depression, anxiety disorder, stroke, Alzheimer’s disease, Parkinson’s disease, and others.

## 5. Conclusion

Our study demonstrated the test–retest reliability of our proposed neuromodulation protocol in reliably modulating brain states. The stimulation targets and parameters were personalized for specific subjects using the brain controllability analysis and the optimal control analysis. The test–retest reliability of the optimal stimulation targets and parameters were evaluated by the ICC measurement. Our results showed that the optimal neuromodulation targets and parameters exhibited excellent reliability, and the model fitting accuracies between the actual final state and the simulated final state displayed good reliability. In conclusion, our work offers empirical support for assessing the test–retest reliability of a currently proposed optimal neuromodulation protocol, providing evidence for future clinical application to reliably locate the optimal stimulation targets and compute the optimal stimulation parameters.

## Data availability statement

Publicly available datasets were analyzed in this study. This data can be found at: http://fcon_1000.projects.nitrc.org/indi/CoRR/html/bnu_1.html.

## Ethics statement

The studies involving human participants were reviewed and approved by State Key Laboratory of Cognitive Neuroscience and Learning at Beijing Normal University. The patients/participants provided their written informed consent to participate in this study.

## Author contributions

FF designed the study, conducted and supervised the data analysis, interpreted the results, drafted the manuscript and also helped with the data collection. JC helped with data analysis and revised the manuscript. RL helped with the interpretation of the results and revised the manuscript. YZ supervised the data analysis, interpreted the results, and revised the manuscript. All authors contributed to the article and approved the submitted version.

## Conflict of interest

The authors declare that the research was conducted in the absence of any commercial or financial relationships that could be construed as a potential conflict of interest.

## Publisher’s note

All claims expressed in this article are solely those of the authors and do not necessarily represent those of their affiliated organizations, or those of the publisher, the editors and the reviewers. Any product that may be evaluated in this article, or claim that may be made by its manufacturer, is not guaranteed or endorsed by the publisher.

## Footnotes

^1^ https://dsi-studio.labsolver.org/

^2^ https://soterixmedical.com/research/hd/mxn-33
